# Robust and accurate prediction of noncoding RNAs from aligned sequences

**DOI:** 10.1186/1471-2105-11-S7-S3

**Published:** 2010-10-15

**Authors:** Yutaka Saito, Kengo Sato, Yasubumi Sakakibara

**Affiliations:** 1 Department of Biosciences and Informatics, Keio University, 3-14-1 Hiyoshi, Kohoku-ku, Yokohama, Kanagawa 223-8522, Japan; 2 Graduate School of Frontier Sciences, University of Tokyo, 5-1-5 Kashiwanoha, Kashiwa, Chiba 277-8561, Japan; 3 Computational Biology Research Center (CBRC), National Institute of Advanced Industrial Science and Technology (AIST), 2-42 Aomi, Koto-ku, Tokyo 135-0064, Japan

## Abstract

**Background:**

Computational prediction of noncoding RNAs (ncRNAs) is an important task in the post-genomic era. One common approach is to utilize the profile information contained in alignment data rather than single sequences. However, this strategy involves the possibility that the quality of input alignments can influence the performance of prediction methods. Therefore, the evaluation of the robustness against alignment errors is necessary as well as the development of accurate prediction methods.

**Results:**

We describe a new method, called Profile BPLA kernel, which predicts ncRNAs from alignment data in combination with support vector machines (SVMs). Profile BPLA kernel is an extension of *base-pairing profile local alignment *(BPLA) kernel which we previously developed for the prediction from single sequences. By utilizing the profile information of alignment data, the proposed kernel can achieve better accuracy than the original BPLA kernel. We show that Profile BPLA kernel outperforms the existing prediction methods which also utilize the profile information using the high-quality structural alignment dataset. In addition to these standard benchmark tests, we extensively evaluate the robustness of Profile BPLA kernel against errors in input alignments. We consider two different types of error: first, that all sequences in an alignment are actually ncRNAs but are aligned ignoring their secondary structures; second, that an alignment contains unrelated sequences which are not ncRNAs but still aligned. In both cases, the effects on the performance of Profile BPLA kernel are surprisingly small. Especially for the latter case, we demonstrate that Profile BPLA kernel is more robust compared to the existing prediction methods.

**Conclusions:**

Profile BPLA kernel provides a promising way for identifying ncRNAs from alignment data. It is more accurate than the existing prediction methods, and can keep its performance under the practical situations in which the quality of input alignments is not necessarily high.

## Background

Reliable identification of noncoding RNA (ncRNA) genes is one of the major goals of recent computational biology [1,2]. In most cases, functional ncRNAs form base-paired secondary structures which are closely related to their roles in organisms. Some algorithms exist for extracting secondary structure information from primary sequences using thermodynamic energy models [3,4]. This information, in addition to nucleotide sequences, can be exploited for the statistical prediction of ncRNAs.

To improve the reliability of predictions, many existing methods take an alignment as input rather than a single sequence [5]. Alignment data provide the profile information of ncRNAs which is not evident from individual sequences; it can help to capture detailed features of primary sequences and secondary structures. Several prediction methods based on support vector machines (SVMs) have been proposed with this respect, and shown to achieve high accuracy [6-8]. Each method has its own kernel function which defines the similarity between a pair of alignment data and determines the accuracy of the SVM classifier. Washietl *et al. *[6] and Gruber *et al. *[7] have developed RNAz, which employs radial basis function (RBF) kernels to compute the similarity of feature vectors of alignment data. A major contribution to its prediction is made by the structure conservation index (SCI) based on thermodynamic energy models. This feature value assesses whether an alignment is structurally conserved by normalizing the minimum free energy of consensus secondary structures with the average of those for individual sequences. Sato *et al. *[8] have developed Profile stem kernel as an extension of Stem kernel which was originally proposed for analyzing single sequences [9]. The method calculates the similarity between a pair of alignment data by summing the substitution scores for all pairs of effective (highly probable) consensus stem structures.

In their studies, input alignments were assumed to be correct or at least not damaging to the accuracy of the prediction methods. However, it is not necessarily the case under the realistic conditions in genomic and transcriptomic screens. Since aligning genomic sequences is an error-prone process [10,11], prediction methods have to deal with low-quality alignment data in most practical applications. For example, RNAz and Profile stem kernel utilize consensus secondary structures as the profile information, which are known to be degraded by the use of low-quality alignment data [12]. The previous studies have not fully evaluated to what extent the quality of input alignments can influence the performance of the prediction methods.

We can consider two different types of error in alignment data: first, that all sequences in an alignment are actually ncRNAs but are aligned ignoring their secondary structures (Type A); second, that an alignment contains unrelated sequences which are not ncRNAs but still aligned (Type B). In the remaining part of this paper, we use these definitions of the Type A and the Type B errors.

The Type A errors are usually involved in genomic and transcriptomic screens since we practically use sequence-based aligners due to the high computational cost for the construction of structural alignment data. In accordance with this convention, the original papers of RNAz and Profile stem kernel tested their methods only on sequence-based alignment datasets [6,8]. On the other hand, some studies have since then attempted to detect ncRNAs from structural alignment data obtained by realigning sequence-based alignments [13,14]. Following these efforts, the recent update of RNAz has reported the results that its accuracy slightly improved when using structural alignment data as input [7]. However, the results were only on the dataset with various ncRNA families mixed, and the improvement for each particular family was not shown. For Profile stem kernel, similar experiments on the Type A errors have not been presented.

The amount of the type B errors has been intensively studied using the 17-way vertebrate alignment in the UCSC genome browser [15]. One study has estimated that 9.7% of the regions include unrelated sequences which are not orthologous to the other sequences in the alignment [10]. More strikingly, the estimate in [11] says that 16% of the segments aligned to ncRNA genes are wrongly included in the alignments from the viewpoint of their secondary structures. In spite of the great significance of the Type B errors suggested by these studies, there has been so far no systematic evaluation about their influence to the performance of prediction methods.

In this paper, we describe a new method, called Profile BPLA kernel, which predicts ncRNAs from alignment data in combination with SVMs. Profile BPLA kernel is an extension of *base-pairing profile local alignment *(BPLA) kernel which we previously developed for the prediction from single sequences [16]. By utilizing the profile information of alignment data, the proposed kernel can achieve better accuracy than the original BPLA kernel. We show that Profile BPLA kernel outperforms the existing prediction methods which also utilize the profile information using the high-quality structural alignment dataset. In addition to these standard benchmark tests, we extensively evaluate the robustness of Profile BPLA kernel against errors in input alignments. For both the Type A and the Type B errors, the effects on the performance of Profile BPLA kernel are surprisingly small. Especially for the Type B errors, we demonstrate that Profile BPLA kernel is more robust compared to the existing prediction methods.

## Results and discussion

### Algorithm

In this section, we propose an accurate and robust method for the prediction of ncRNAs from alignment data. The proposed method, named Profile BPLA kernel, is an extension of BPLA kernel which we previously developed for the prediction from single sequences [16]. Hence, we first review the original algorithm of BPLA kernel, and then extend the method to alignment data.

The whole schemes of the original BPLA kernel and Profile BPLA kernel are summarized in Figure S1 (Additional file 1).

#### Notations

For an RNA sequence **x**, we denote its length by |**x**|, and the nucleotide at the *i*-th position by *x_i_*. For a pair of sequences, **x **and **y**, we denote the set of all possible local alignments in the Smith-Waterman (SW) algorithm [17] by Π**_xy_**, and one particular local alignment in Π**_xy _**by *π***_xy_**. We denote the alignment score of *π***_xy _**by Score(*π***_xy_**), which is calculated based on a scoring function *S***_xy_**(*i*, *j*) for matching the *i*-th position in **x **and the *j*-th position in **y**. We design *S***_xy_**(*i*, *j*) using a nucleotide substitution matrix *s*(*x*_*i*, _*y*_*j*_) as its component. In addition, we use four parameters: *α*, *β*, *g*, and *d*.

For each sequence **x**, we denote the set of all possible secondary structures by Θ**_x_**, and one particular secondary structure in Θ**_x _**by *θ***_x_**. We represent a secondary structure by *θ***_x _**= {*θ***_x_**(*i*, *j*)}*_i < j_*, where a binary variable *θ***_x_**(*i*, *j*) is equal to one only when the *i*-th position and the *j*-th position in **x **form a base pair. In addition, for each position *i *in **x**, we define three kinds of binary variable: *L***_x_**(*i*) = Σ_*j*:*j *>*i *_*θ***_x_**(*i*, *j*) is equal to one only when a pair is formed with one of the downstream positions; *R***_x_**(*i*) = Σ_*j*:*j *<*i *_*θ***_x_**(*j*, *i*) is equal to one only when a pair is formed with one of the upstream positions; and *U***_x_**(*i*) = 1 - *L***_x_**(*i*) - *R***_x_**(*i*) is equal to one only when the position is unpaired. These binary variables are converted to the corresponding probabilities by taking the expectation over Θ**_x_**. For *θ***_x_**(*i*, *j*), we obtain a base-pairing probability matrix, which consists of the probabilities *P***_x_**(*i*, *j*) that the *i*-th and the *j*-th positions form a base pair:

$$P_{x}(i,j)=\sum_{\theta_{x}\in \Theta_{x}}\theta_{x}\left(i,j\right)P\left(\theta_{x}|x\right),$$

where the probability distribution *P*(*θ***_x_**|x) is computed with the McCaskill algorithm [4] based on thermodynamic energy models. For {*L***_x_**(*i*), *R***_x_**(*i*), *U***_x_**(*i*)}, we obtain a *base-pairing profile *[18], which consists of the probabilities {*P***_x_***^L^*(*i*), *P***_x_***^R^*(*i*), *P***_x_***^U ^*(*i*)} that the *i*-th position is paired with one of the downstream/upstream positions, or unpaired, respectively:

$$\begin{array}{l} P_{x}^{L}(i)\;=\;\sum_{\theta_{x}\in \Theta_{x}}L_{x}(i)P(\theta_{x}|x)=\sum_{\theta_{\text{x}}\in \Theta_{\text{x}}}\sum_{j:j>i}\theta_{x}(i,j)P(\theta_{x}|x)=\sum_{j:j>i}P_{x}(i,j),\\ P_{x}^{R}(i)\;=\;\sum_{\theta_{x}\in \Theta_{x}}R_{x}(i)P(\theta_{x}|x)=\sum_{\theta_{x}\in \Theta_{x}}\sum_{j:j<i}\theta_{x}(j,i)P(\theta_{x}|x)=\sum_{j:j<i}P_{x}(j,i),\\ P_{x}^{U}(i)\;=\;\sum_{\theta_{x}\in \Theta_{x}}U_{x}(i)P(\theta_{x}|x)=1-P_{x}^{L}(i)-P_{x}^{R}(i). \end{array}$$

For a multiple alignment **X**, we denote the *i*-th column by *X_i_*, and the *k*-th sequence by **X***^k^*. The nucleotide at the *i*-th position in **X***^k ^*is denoted by $X_{i}^{k}$, which can be a gap character.

#### Original BPLA kernel for single sequences

A kernel function is a measure of similarity between a pair of objects and can be used as a prediction method in combination with an SVM classifier as long as Mercer's condition is satisfied [19]. BPLA kernel calculates the similarity between a pair of RNA sequences using the information of their primary sequences and secondary structures.

The basic idea of BPLA kernel is to perform a pairwise alignment and then to regard the alignment score as the measure of similarity. Instead of relying on one optimal alignment, we accumulate the scores of all possible local alignments in the SW algorithm using *local alignment *(LA) kernel [20]. LA kernel between two sequences, **x **and **y**, is defined as follows:

(1) $$K(x,y)=\sum_{\pi_{xy}\in \Pi_{xy}}e^{\beta \text{Score}(\pi_{xy})},$$

where *β *≥ 0 is a concentration parameter. Given a scoring function *S***_xy_**(*i*, *j*) for the alignment score Score(*π***_xy_**), LA kernel (1) can be computed by the following algorithm:

Initialization:

**for ***i *∈ {0, ..., |**x**|} and *j *∈ {0, ..., |**y**|} **do**

   *M*(*i*, 0) = *I_X _*(*i*, 0) = *I_Y _*(*i*, 0) = *T_X _*(*i*, 0) = *T_Y _*(*i*, 0) = 0

   *M*(0, *j*) = *I_X _*(0, *j*) = *I_Y _*(0, *j*) = *T_X _*(0, *j*) = *T_Y _*(0, *j*) = 0

end for

Iteration:

**for ***i ∈* {1, ..., |**x**|} and *j *∈ {1, ..., |**y**|} **do**

   *M*(*i*, *j*) = *e*^*βS***xy**(*i,j*)^(1 + *I_X _*(*i - *1, *j - *1) + *I_Y _*(*i - *1, *j - *1) + *M*(*i - *1, *j - *1))

   *I_X _*(*i*, *j*) = *e^βg^M*(*i - *1, *j*) + *e^βd^I_X _*(*i - *1, *j*)

   *I_Y _*(*i*, *j*) = *e^βg^*(*M*(*i*, *j - *1) + *I_X _*(*i*, *j - *1)) + *e^βd^I_Y _*(*i*, *j - *1)

   *T_X _*(*i*, *j*) = *M*(*i - *1, *j*) + *T_X _*(*i - *1, *j*)

   *T_Y _*(*i*, *j*) = *M*(*i*, *j - *1) + *T_X _*(*i*, *j - *1) + *T_Y _*(*i*, *j - *1)

end for

Termination:

*K*(**x**, **y**) = 1 + *T_X _*(|**x**|, |**y**|) + *T_Y _*(|**x**|, |**y**|) + *M*(|**x**|, |**y**|)

where the parameters *g *and *d *are penalties for gap opening and gap extension, respectively. In practice, kernel values are normalized to range from 0 to 1:

$$K_{n}(x,y)=\frac{K(x,y)}{\sqrt{K(x,x)K(y,y)}}.$$

To incorporate secondary structure information into the match score *S***_xy_**(*i*, *j*), we employ the scoring function used in STRAL [21]. For each sequence **x**, we first compute a base-pairing probability matrix *P***_x_**(*i*, *j*) using the Vienna RNA package [22] which is an implementation of the McCaskill algorithm. Subsequently, for each position *i *in **x**, we summarize the base-pairing probabilities into the base-pairing profile $\left\{P_{x}^{L}\left(i\right),P_{x}^{R}\left(i\right),P_{x}^{U}\left(i\right)\right\}$. We define the scoring function *S***_xy_**(*i, j*) using the base-pairing profiles as follows:

(2) $$\begin{array}{l} S_{xy}(i,j)\;=\;\alpha S_{\text{struct}}+S_{\text{seq}}\\ \;\;\;\;\;\;\;\;\;\;\;\;=\alpha \left(\sqrt{P_{x}^{L}(i)P_{y}^{L}(j)}+\sqrt{P_{x}^{R}(i)P_{y}^{R}(j)}\right)+s(x_{i},y_{j})\sqrt{P_{x}^{U}(i)P_{y}^{U}(j)}, \end{array}$$

where *α *≥ 0 is a weight parameter for structural information, and a nucleotide substitution score *s*(*x_i_*, *y_j_*) captures the similarity of primary sequences. We use the RIBOSUM 85-60 substitution matrix [23] as *s*(*x_i_*, *y_j_*) with the minor modification that its smallest eigenvalue is subtracted from each of its diagonal elements in order to satisfy Mercer's condition.

Combining LA kernel (1) with the scoring function (2), we call this method *base-pairing profile local alignment *(BPLA) kernel.

#### Profile BPLA kernel for alignment data

Now we extend BPLA kernel to the prediction from alignment data. Profile BPLA kernel for alignment data need to define the similarity between a pair of alignment data instead of a pair of single sequences. More specifically, the new algorithm needs to perform pairwise alignments between two alignment data, and calculate their alignment scores. This problem reduces to the definition of a scoring function corresponding to (2) for two alignment columns instead of two sequence positions. Both *S*_struct _and *S*_seq _in (2) should be extended to take into account the profile information contained in the alignment columns. In order to define the structural similarity *S*_struct _between two alignment columns, we need a base-pairing profile for each alignment column. This can be calculated if we define a base-pairing probability matrix for a multiple alignment. As shown in [12,24], the consensus secondary structures of aligned sequences are accurately modeled by averaging the individual base-pairing probability matrices. Thus, we define a base-pairing probability matrix for a multiple alignment **X **as follows:

$$\begin{array}{l} P_{X}(i,j)\;=\;\frac{1}{N(X)}\sum_{k=1}^{N(X)}{P^{\prime }}_{X^{k}}(i,j),\\ {P^{\prime }}_{X^{k}}(i,j)=\{\begin{array}{ll} P_{X^{k^{\prime }}}(r(i),r(j)) & \left(\text{either}\;\text{of}\;X_{i}^{k}\;\text{or}\;X_{j}^{k}\;\text{is}\;\text{not}\;\text{a}\;\text{gap}\right)\\ 0 & (\text{otherwise}), \end{array} \end{array}$$

where **X***^k' ^*is the original sequence of **X***^k ^*without gaps, *r*(*i*) is the index in **X***^k' ^*corresponding to the *i*-th position in **X***^k^*, and *N*(**X**) is the number of aligned sequences in **X**.

The sequence similarity *S*_seq _can be extended by defining a substitution score *s*(·,·) between two alignment columns. We use the averaged score of all possible substitutions between two columns, *X_i _*and *Y_j_*:

$$\begin{array}{l} s(X_{i},Y_{j})\;=\;\frac{1}{N(X)N(Y)}\sum_{k=1}^{N(X)}\sum_{l=1}^{N(Y)}s^{\prime }(X_{i}^{k},Y_{j}^{l}),\\ s^{\prime }(X_{i}^{k},Y_{j}^{l})=\{\begin{array}{ll} s(X_{i}^{k},Y_{j}^{l}) & \left(\text{either}\;\text{of}\;X_{i}^{k}\;\text{or}\;Y_{j}^{l}\;\text{is}\;\text{not}\;\text{a}\;\text{gap}\right)\\ 0 & (\text{otherwise}). \end{array} \end{array}$$

This is equivalent to the sum-of-pairs score, which is widely used in the problem of group-to-group alignment for primary sequences.

#### Rationale for the scoring function

Although the scoring function (2) in our method has been first proposed for STRAL, its theoretical aspect has been not fully clarified in the previous study [21]. Here, we formulate the scoring function (2) in the different manner from [21]. For this purpose, let us consider the following scoring function.

(3) $$W_{xy}\left(i,j|\theta_{x}{}_{,}\theta_{y}\right)\;=\alpha \left(L_{x}\left(i\right)L_{y}\left(j\right)\;+R_{x}\left(i\right)R_{y}\left(j\right)\right)\;+s\left(x_{i},y_{j}\right)U_{x}\left(i\right)U_{y}\left(j\right).$$

Given a pair of secondary structures, *θ***_x _**for **x **and *θ***_y _**for **y**, this function defines the score for matching the *i*-th position in **x **and the *j*-th position in **y**. The score can take a non-zero value in three different cases depending on the direction of base-pairing at the *i*-th position in *θ***_x _**and the *j*-th position in *θ***_y_**: it takes α when both of the two positions form a base pair with one of their downstream positions, respectively; it takes α when both of the two positions form a base pair with one of their upstream positions, respectively; and it takes *s*(*x_i_*, *y_j_*) when both of the two positions are unpaired. Thus, the scoring function (3) evaluates the similarity based on the criteria of whether the two positions have the same state of base-pairing.

In the equation (3), we need one fixed pair of secondary structures, *θ***_x _**and *θ***_y_**. However, we typically do not know one reliable secondary structure for each of **x **and **y**, and have the uncertainty about many suboptimal secondary structures. Therefore, we use the ensemble of all possible secondary structures by taking the expectation of (3) over Θ**_x _**and Θ**_y_**:

(4) $$\sum_{\theta_{\text{x}}\in \Theta_{\text{x}}}\sum_{\theta_{\text{y}}\in \Theta_{\text{y}}}W_{\text{xy}}(i,j|\theta_{\text{x}},\theta_{\text{y}})P(\theta_{\text{x}}|\text{x})P(\theta_{\text{y}}|\text{y})=\alpha (P_{\text{x}}^{L}(i)P_{\text{y}}^{L}(j)+P_{\text{x}}^{R}(i)P_{\text{y}}^{R}(j))+s(x_{i},y_{j})P_{\text{x}}^{U}(i)P_{\text{y}}^{U}(j).$$.

The resulting scoring function (4) can be regarded as a variant of the STRAL's scoring function (2) without square-root operations. In practice, we find that (2) gives slightly better performance compared to (4), and thus use (2) for the component of our method.

### Performance evaluation

In this section, we examine the accuracy of Profile BPLA kernel in comparison to the state-of-the-art prediction methods based on SVMs. Furthermore, we present a systematic evaluation about the robustness of Profile BPLA kernel against the Type A and the Type B errors in input alignments. See Background for the definitions of the Type A and the Type B errors.

#### Dataset and experimental system

We created a dataset which includes 990 positive samples from five ncRNA families: C/D snoRNAs, H/ACA snoRNAs, miRNA precursors, riboswitches, and tRNAs. These families were collected by combining 885 smaller families in the Rfam database [25] into larger categories (Table 1). Each positive sample is an alignment of ncRNAs, and is separated by a sequence identity of less than 60% from the other alignment data (see Methods for details). For the construction of input alignments, we produced two versions of the dataset: the high-quality structural alignments by RAF [26], and the sequence-based alignments by CLUSTALW [27]. We generated negative samples which have the same dinucleotide contents as the positives using the randomization by SISSIz [28].

**Table 1:** Summary of the combined Rfam families.

Family	NF	N	NS
C/D snoRNA	340	272	5
H/ACA snoRNA	133	119	5
miRNA precursor	401	431	5
Riboswitch	10	85	3
tRNA	1	83	3

Family: name of the larger category used in the performance evaluation. NF: number of smaller families in the Rfam database which were combined. N: number of positive samples. NS: average number of aligned sequences per sample.

The accuracy of the prediction methods was assessed by the area under the receiver operating characteristic (ROC) curve, *i.e*., the ROC score. The ROC curve plots the true positive rate *TP/*(*TP *+ *FN*) versus false positive rate *FP/*(*TN *+ *FP *) for different decision thresholds of a SVM classifier, where *TP *is the number of correctly predicted positives, *FP *is the number of incorrectly predicted positives, *TN *is the number of correctly predicted negatives, and *FN *is the number of incorrectly predicted negatives. We used four-fold cross-validation with the following modifications. The SVM classifier was trained with the same number of negative samples as the positives, and tested on a data partition which includes eight times as many negative samples as the positives. This problem setting is analogous to genomic and transcriptomic screens, where the vast majority of the search space does not contain functional ncRNA genes. Moreover, the four-fold cross validation is repeated four times with different splits of the dataset (16 trials in total). The parameters *α*, *β*, *g*, and *d *in Profile BPLA kernel were adapted to the training data using the gradient-based optimization developed for the original BPLA kernel [29]. Note that we did not used the test data for the parameter optimization to avoid overfitting.

#### Accuracy improvement by the profile information

We first examined whether the proposed kernel could achieve better accuracy than the original BPLA kernel by utilizing the profile information of alignment data. For this purpose, the dataset of single sequences was created from the alignment dataset described above. For positive samples, we randomly chose one sequence from each alignment of ncRNAs. We generated negative samples which have the same dinucleotide contents as the positives by the standard shuffling procedure [30]. Then, the proposed kernel and the original BPLA kernel were compared using the high-quality structural alignment dataset and the corresponding single sequence dataset, respectively.

Table 2 presents the experimental results. As expected, the proposed kernel achieved the better ROC scores than the original BPLA kernel for the all ncRNA families. These results suggest that the profile information contained in alignment data is useful to improve the prediction of ncRNAs.

**Table 2:** Accuracy improvement by the profile information.

	ROC score (stdev)	
Family	Original BPLA kernel	Profile BPLA kernel
C/D snoRNA	0.91 (0.02)	0.95 (0.02)
H/ACA snoRNA	0.93 (0.03)	0.97 (0.02)
miRNA precursor	0.96 (0.01)	0.97 (0.01)
Riboswitch	0.86 (0.04)	0.92 (0.05)
tRNA	0.98 (0.02)	1.00 (0.00)

average	0.93 (0.02)	0.96 (0.02)

Family: name of the target ncRNA family. ROC score: area under the ROC curve. Profile BPLA kernel, which utilizes the profile information of alignment data, is compared to the original BPLA kernel for single sequences.

#### Accuracy on the high-quality structural alignment dataset

Next, we compared Profile BPLA kernel with the existing prediction methods which also utilize the profile information. In the ideal condition, the profile information should be extracted from high-quality alignment data such that all sequences are actually ncRNAs and aligned taking into account their secondary structures. Therefore, we tested the accuracy of each prediction method using the high-quality structural alignment dataset constructed by RAF. The competitors were RNAz [6,7] and Profile stem kernel [8]. We also performed the experiment with the profile version of LA kernel, which does not consider secondary structure information, by setting base-pairing profiles {*L***_x_**(*i*) = 0, *R***_x_**(*i*) = 0, *U***_x_**(*i*) = 1} in Profile BPLA kernel.

Table 3 presents the experimental results. Profile BPLA kernel outperformed the other prediction methods except for riboswitches, and achieved the best ROC score on average. The accuracy of Profile LA kernel was severely limited compared to the prediction methods which consider secondary structure information. However, for C/D snoRNAs, Profile LA kernel resulted in the comparable ROC score with RNAz and Profile stem kernel. These results suggest that RNAz and Profile stem kernel may fail to incorporate the effective information of secondary structures. Profile BPLA kernel consistently achieved the better ROC scores than Profile LA kernel, showing its wide applicability.

**Table 3:** Accuracy on the high-quality structural alignment dataset.

	ROC score (stdev)			
Family	Profile BPLA kernel	Profile LA kernel	Profile stem kernel	RNAz
C/D snoRNA	0.95 (0.02)	0.79 (0.04)	0.80 (0.02)	0.78 (0.03)
H/ACA snoRNA	0.97 (0.02)	0.65 (0.20)	0.89 (0.04)	0.95 (0.03)
miRNA precursor	0.97 (0.01)	0.69 (0.02)	0.92 (0.01)	0.96 (0.01)
Riboswitch	0.92 (0.05)	0.41 (0.23)	0.77 (0.05)	0.97 (0.02)
tRNA	1.00 (0.00)	0.88 (0.03)	0.95 (0.02)	0.96 (0.02)

Average	0.96 (0.02)	0.69 (0.10)	0.86 (0.03)	0.92 (0.02)

Family: name of the target ncRNA family. ROC score: area under the ROC curve. Profile BPLA kernel is compared to the other prediction methods which also utilize the profile information of alignment data: Profile LA kernel, Profile stem kernel, and RNAz.

The superiority of Profile BPLA kernel is inherited from the original BPLA kernel. In our previous paper [16], we have proved that the original BPLA kernel outperforms the non-profile versions of Stem kernel and LA kernel. Our results showed the high accuracy of BPLA kernels in the prediction from alignment data as well as from single sequences. (Note that the non-profile version of RNAz does not exist since the feature values of alignment data used in the method can not be defined for single sequences.)

#### Robustness against the Type A errors

In addition to the standard benchmark tests, we extensively evaluated the robustness of Profile BPLA kernel against errors in input alignments. To discuss the Type A errors, we performed the experiment using the sequence-based alignment dataset constructed by CLUSTALW instead of the high-quality structural alignment dataset.

By comparing the results in Table 4 with those in Table 3, we can see the robustness of each prediction method against the Type A errors. Profile BPLA kernel achieved almost the same ROC scores for the two datasets, showing the comparable robustness to RNAz and Profile stem kernel.

**Table 4:** Accuracy on the sequence-based alignment dataset.

	ROC score (stdev)			
Family	Profile BPLA kernel	Profile LA kernel	Profile stem kernel	RNAz
C/D snoRNA	0.95 (0.01)	0.80 (0.04)	0.80 (0.02)	0.77 (0.02)
H/ACA snoRNA	0.96 (0.02)	0.77 (0.17)	0.87 (0.03)	0.94 (0.03)
miRNA precursor	0.97 (0.01)	0.69 (0.03)	0.92 (0.02)	0.96 (0.01)
Riboswitch	0.92 (0.03)	0.38 (0.19)	0.79 (0.05)	0.94 (0.02)
tRNA	1.00 (0.00)	0.88 (0.03)	0.94 (0.03)	0.95 (0.02)

average	0.96 (0.02)	0.70 (0.09)	0.86 (0.03)	0.91 (0.02)

Family: name of the target ncRNA family. ROC score: area under the ROC curve. Profile BPLA kernel is compared to the other prediction methods which also utilize the profile information of alignment data: Profile LA kernel, Profile stem kernel, and RNAz.

The robustness of Profile BPLA kernel can be attributed to its formulation. Profile BPLA kernel utilizes averaged base-pairing probability matrices to obtain the profile information of secondary structures. Averaged base-pairing probability matrices have been shown to be useful for the robust modeling of consensus secondary structures against the Type A errors [12]. Our results showed the effectiveness of averaging base-pairing probabilities for the robustness in the problem of ncRNA prediction.

Our experiment provided the detailed evaluation of the robustness for each particular ncRNA family. The recent study has reported that the accuracy of RNAz can be slightly improved by the use of structural alignment data [7]. However, the experiment in [7] has been performed on the dataset with various families mixed. In our experiment, we found that the Type A errors had different effects on the performance of each prediction method depending on families. This in-depth view of the robustness is especially important when we target a particular family in genomic and transcriptomic screens.

Our results also demonstrated that Profile BPLA kernel outperformed the existing prediction methods in the "realistic" condition considered in the previous studies [6-8]. Profile BPLA kernel achieved the best ROC scores for the sequence-based alignment dataset with the Type A errors as well as for the high-quality structural alignment dataset. In the following experiments, we further evaluated the robustness of Profile BPLA kernel against the Type B errors which have been neglected in the previous studies.

#### Robustness against the Type B errors

For the systematic evaluation of the robustness, we prepared a controlled series of alignment data with different degrees of the Type B errors. Input alignments in genomic and transcriptomic screens are typically constructed by sequence-based alignment tools. Hence, alignment data with the Type B errors are expected to be optimal at least under the criteria of sequence-based alignment tools, even though incorrect from the viewpoint of secondary structures. Based on this assumption, we generated sequences which can be well aligned to a given alignment in terms of primary sequences, but do not conserve its consensus secondary structure (see Methods for details). By introducing these "unrelated" sequences, we simulated the Type B errors in the sequence-based alignment dataset. For each positive sample in the test data, a series of erroneous alignments was prepared by gradually replacing ncRNA sequences with unrelated sequences. We aligned the unrelated sequences with the remaining ncRNA sequences using CLUSTALW. The resulting alignments were then used to make the equal-size datasets for the different fractions of unrelated sequences ranged from 0.0 to 1.0 at intervals of 0.1. An alignment comprising *n *ncRNA sequences and *m *unrelated sequences was included in the dataset of the fraction *f *satisfying (*m - *1)/(*n *+ *m*) < f ≤ *m/*(*n *+ *m*). We trained the SVM classifiers with the original training data in the sequence-based alignment dataset, and tested them on the datasets with the different degrees of the simulated Type B errors. The performance was assessed by the ROC score for discriminating the erroneous alignments from the alignments consisting only of unrelated sequences.

**Figure 1:**
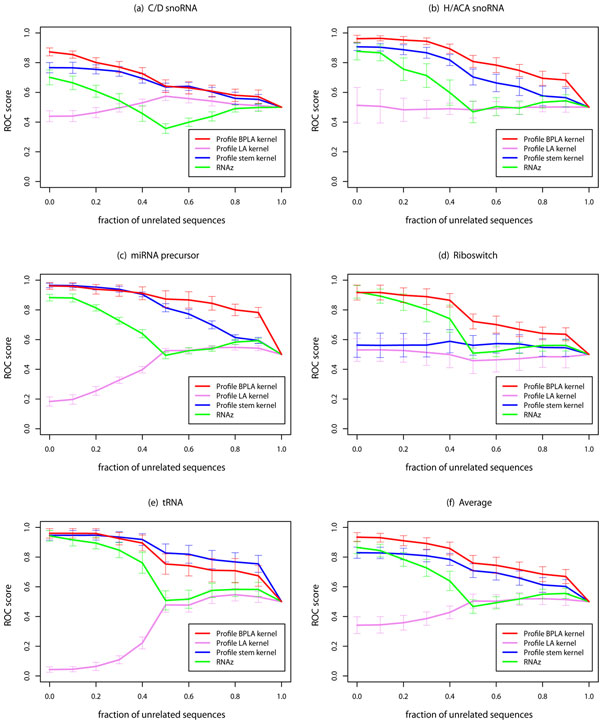
**Accuracy on the sequence-based alignment dataset with different fractions of unrelated sequences**. For each point, the alignments with the different fraction of unrelated sequences were discriminated from the negative samples which consist only of unrelated sequences. Zero in the horizontal axis corresponds to the detection of the alignments which consist only of actual ncRNAs, *i.e*., an ordinary discrimination problem without the Type B errors. The error bars show standard deviation of ROC scores.

The experimental results are shown in Figure 1. In this figure, zero in the horizontal axis is equivalent to an ordinary prediction problem in which alignments to be discriminated from negative samples do not contain any unrelated sequences. In this situation, Profile BPLA kernel achieved the best accuracy on average, being consistent with the results in Table 4. (The ROC scores, however, were not exactly the same as those in Table 4 since we used the different kind of negative samples in the test data between the two experiments: alignments consisting only of unrelated sequences for Figure 1, and dinucleotide-controlled samples for Table 4.) As the fraction of unrelated sequences increased, the ROC scores for RNAz rapidly fell down to the baseline. In contrast, Profile BPLA kernel kept the discrimination at high levels until the alignments were overwhelmed by the Type B errors. A similar tendency was seen for Profile stem kernel, although its ROC scores were smaller than Profile BPLA kernel. The performance of Profile LA kernel was seriously damaged by the Type B errors since the method does not consider secondary structures of unrelated sequences. These results suggest that Profile BPLA kernel is the only method which can effectively detect ncRNAs in the presence of the Type B errors.

**Figure 2:**
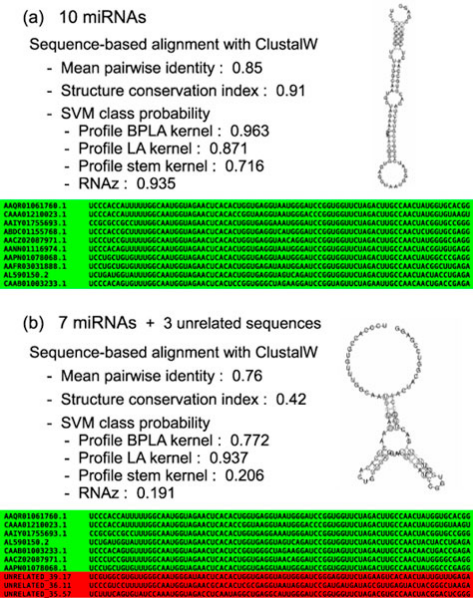
**Example of the Type B errors and its influence on the prediction methods**. (a) Native alignment consisting only of ncRNAs. An alignment of 10 miRNA precursors is highly conserved in terms of both primary sequences and secondary structures. The consensus secondary structure predicted by RNAalifold [33] exhibits a well-known hairpin loop. Profile BPLA kernel and the other prediction methods accepted this alignment. (b) Alignment with the Type B errors. Three miRNA precursors in the native alignment were replaced with unrelated sequences, which destroyed the consensus secondary structure. This alignment was rejected by RNAz due to the drastic decrease in the SCI and also missed by Profile stem kernel. Profile LA kernel was completely ruined showing the higher SVM class probability for the erroneous alignment than that for the native one. Profile BPLA kernel was the only method to accept the alignment by the moderate decrease in the SVM class probability from the native one. Note that the mean pairwise identity is still high allowing this alignment to be produced by sequence-based alignment tools.

The observed differences in the robustness among the methods are deeply connected with the rationales behind their predictions. RNAz detects ncRNAs by utilizing the SCI which measures the conservation of secondary structures in an alignment. Therefore, the experimental results for RNAz can be interpreted as showing that unrelated sequences cause noise in a conserved secondary structure. Profile BPLA kernel do not measure the conservation of secondary structures. Instead, we directly calculate the similarity of secondary structures between input alignments and training data. Hence, Profile BPLA kernel can detect an alignment containing only a few ncRNA sequences if they are similar enough to the ncRNAs in training data, even though the alignment itself is not structurally conserved. Figure 2 illustrates an example of the Type B errors and its influences on the performance of the prediction methods. Although RNAz accepted the native alignment (Figure 2a), it rejected the erroneous alignment (Figure 2b) due to the drastic decrease in the SCI value. On the other hand, Profile BPLA kernel kept the SVM class probability moderate for the erroneous alignment, accepting the seven miRNA precursors included in the alignment. Note that the erroneous alignment in Figure 2b can be regarded as conserved if we focus only on the sequence identity. This suggests that such alignments can be produced by most alignment tools which do not consider secondary structures. In fact, several studies have suggested that genomic alignments contain significant amounts of the Type B errors [10,11]. Therefore, the robustness of Profile BPLA kernel is a desirable characteristic for practical applications.

**Figure 3:**
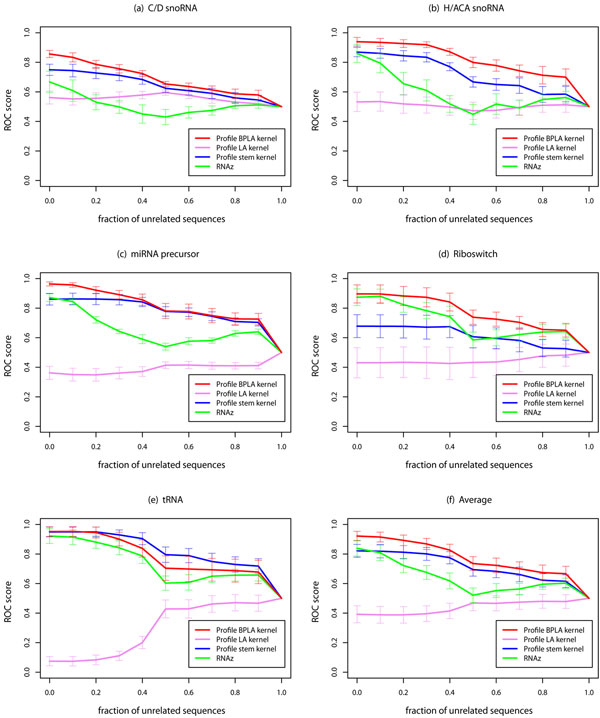
**Accuracy on the structural alignment dataset with different fractions of unrelated sequences**. For each point, the alignments with the different fraction of unrelated sequences were discriminated from the negative samples which consist only of unrelated sequences. Zero in the horizontal axis corresponds to the detection of the alignments which consist only of actual ncRNAs, *i.e*., an ordinary discrimination problem without the Type B errors. The error bars show standard deviation of ROC scores.

**Figure 4:**
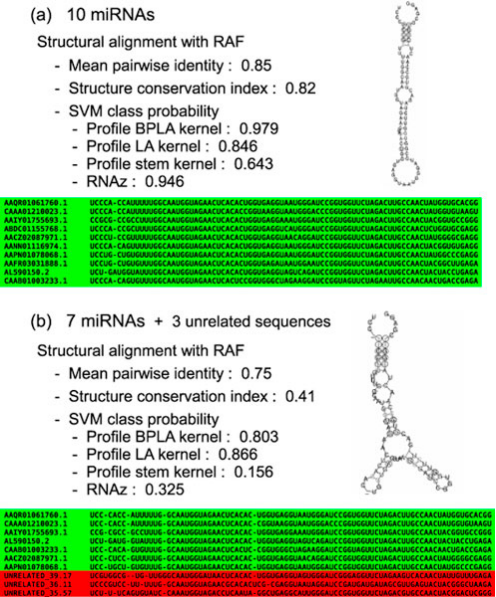
**Realigning unrelated sequences by structural alignment tools attempting to correct the Type B errors**. (a) Native alignment consisting only of ncRNAs. (b) Alignment with the Type B errors. In contrast to the type A errors, the Type B errors cannot be corrected even if we realign the alignments using structural alignment tools. Profile BPLA kernel was still the only method to accept the seven miRNA precursors in the alignment with the Type B errors.

We emphasize that the Type B errors can not be corrected even if we realign the alignments using structural alignment tools as attempted in [13,14]. In contrast to the Type A errors, the Type B errors are caused by the inclusion of unrelated sequences rather than the small shifts of matches and gaps. To make this point clear, we performed the same experiment as in Figure 1 and Figure 2 using RAF instead of CLUSTALW. For the training data, we used the high-quality structural alignment dataset, and for the test data, we used the erroneous alignment realigned by RAF. As expected, the results in Figure 3 and Figure 4 were close to those in Figure 1 and Figure 2, respectively. In Figure 3, Profile BPLA kernel outperformed the existing prediction methods for native alignments, and successfully kept the discrimination for alignments with moderate degrees of the Type B errors. Although the erroneous alignment in Figure 4b was slightly changed from that in Figure 2b, the outputs of the prediction methods were not significantly improved. These results suggest that the problem of the Type B errors is inevitable, and the robustness of Profile BPLA kernel is essential to detect ncRNAs from low-quality alignment data.

## Conclusions

We have described a new method for the prediction of ncRNAs from alignment data. Our method, named Profile BPLA kernel, is an extension of BPLA kernel which was originally developed for the prediction from single sequences [16]. By utilizing the profile information of alignment data, the proposed kernel can achieve better accuracy than the original BPLA kernel. Furthermore, Profile BPLA kernel outperforms the state-of-the-art prediction methods [6-8] which also utilize the profile information.

The evaluation of the robustness against errors in input alignments is a crucial step for the development of practical prediction methods. Even with prediction methods showing excellent accuracy for well-curated alignment datasets, the same performance typically cannot be expected in the practical situations which involve significant amounts of alignment errors. Previous studies did not fully address this issue. Through the present study, we extensively evaluated the effectiveness of Profile BPLA kernel under the realistic conditions in which the quality of input alignments is not necessarily high. We considered the two different types of error in alignment data: first, that all sequences in an alignment are actually ncRNAs but are aligned ignoring their secondary structures (Type A); second, that an alignment contains unrelated sequences which are not ncRNAs but still aligned (Type B). Our experiments presented the more detailed evaluation for the Type A errors than the previous study [7], and the first systematic evaluation for the Type B errors. For the Type A errors, Profile BPLA kernel has the comparable robustness to the existing prediction methods. For the Type B errors, Profile BPLA kernel achieves the higher level of robustness than the existing prediction methods.

We conclude that Profile BPLA kernel provides a promising way for identifying ncRNAs genes from alignment data.

## Methods

### Combining related Rfam families

We created the datasets for the benchmark tests using the Rfam database [25] version 9.1. To make the tests more challenging, we combined related Rfam families into larger categories as shown in Table 1. For example, the C/D snoRNA family in Table 1 was established by combining the 340 Rfam families which have the string "snoRNA; CD-box;" in the description track. The seed alignments for these families were then split into single sequences. We performed a complete linkage clustering using their sequence identity as the similarity function. Clusters were determined using the similarity threshold of 60%, and we obtained one alignment from each cluster consisting of multiple sequences.

### Generating unrelated sequences

We generated unrelated sequences for simulating the Type B errors in alignment data. For each larger category in Table 1, we took the seed alignments of the corresponding smaller Rfam families. For each seed alignment, we constructed a profile hidden Markov model (profile HMM) using HMMER [31], and a covariance model (CM) using INFERNAL [32]. Profile HMMs and CMs are grammar models to generate sequences which can be well aligned to given alignments, and to calculate scores for aligning generated sequences to the original alignments. Profile HMMs do not consider the constraints of consensus secondary structures in alignments, whereas CMs do. We generated 100000 sequences from the profile HMM, and calculated the scores for aligning these sequences using the profile HMM and the CM. We needed sequences which can be well aligned to a given alignment, but do not conserve its consensus secondary structure. Therefore, we chose the top 100 sequences whose score difference between the profile HMM and the CM was large, and used them as the pool of unrelated sequences.

### Software versions and options

We used the most recent version of each software, and if not specified, executed it with the default options. We used RNAz [6,7] version 2.0 and Profile stem kernel [8] version 216c. For the computation of base-pairing probability matrices, we used the Vienna RNA package [22] version 1.8.4. To construct the sequence-based and the structural alignment datasets, we used CLUSTALW [27] version 1.83 and RAF [26] version 1.00, respectively. To generate the negative samples, we used SISSIZ version 0.1 with the option "--simulate --tstv --precision 0.05 --rna" recommended in the original paper [28]. For the prediction of the consensus secondary structures shown in Figure 2 and Figure 4, we used RNAalifold [33] included in the Vienna RNA package version 1.8.4. To simulate the unrelated sequences for the Type B errors, we used the HMMER package [31] version 2.3.2 and the INFERNAL package [32] version 1.0. For the individual programs in the HMMER and the INFERNAL packages, we used the following commands: "hmmbuild -g", "hmmsearch -E 100000", and "cmsearch -g -T -10000 --toponly --no-qdb --fil-no-hmm --fil-no-qdb". Basically, these options were set because we needed global alignments rather than local alignments for the evaluation of the Type B errors, and wanted to calculate the exact scores for profile HMMs and CMs without several heuristics implemented in the programs.

### Availability

Our implementation of Profile BPLA kernel (including the original BPLA kernel for single sequences) is freely available at http://bpla-kernel.dna.bio.keio.ac.jp/ under the GNU general public license. It takes RNA sequences or multiple alignments, and calculates a kernel matrix, which can be used as an input for a popular SVM tool called LIBSVM [34]. Furthermore, our software is capable of parallel processing using the message passing interface (MPI) [35].

## Competing interests

The authors declare that they have no competing interests.

## Authors' contributions

Y Saito extended the code, performed the experiments and drafted the manuscript. KS developed the algorithm and wrote the original code. Y Sakakibara conceived of the study, and participated in its design and coordination. All authors have read and approved the final manuscript.

## Supplementary Material

Additional file 1 **Figure S1**. Overview of the original BPLA kernel and Profile BPLA kernel. The whole schemes of our method were summarized using a pseudo-code in PDF format. Click here for file
