# Supplementary material for: Robust and accurate prediction of noncoding RNAs from aligned sequences
Source: BMC Bioinformatics. 2010 Oct 15;11(Suppl 7):S3. doi: 10.1186/1471-2105-11-S7-S3 (PMC2957686; doi:10.1186/1471-2105-11-S7-S3)
Supplement: Additional file 1 — Figure S1. Overview of the original BPLA kernel and Profile BPLA kernel. The whole schemes of our method were summarized using a pseudo-code in PDF format. [file 1471-2105-11-S7-S3-S1.pdf]

(a) Original BPLA kernel

```
INPUT:
training data (set of RNA sequences)
test data (set of RNA sequences)

OUTPUT:
SVM class probability for each of test data


(1) Training

for each sequence x in training data
  compute a base-pairing probability matrix P_x;
  for each position i in x
    compute a base-pairing profile {P_x^L(i), P_x^R(i), P_x^U(i)};
  end for
end for

for each sequence x in training data
  for each sequence y in training data
    compute a value of BPLA kernel K^train(x,y);
  end for
end for

Train a SVM classifier using K^train;


(2) Test

for each sequence x in test data
  compute a base-pairing probability matrix P_x;
  for each position i in x
    compute a base-pairing profile {P_x^L(i), P_x^R(i), P_x^U(i)};
  end for
end for

for each sequence x in test data
  for each sequence y in training data
    compute a value of BPLA kernel K^test(x,y);
  end for
end for

for each sequence x in test data
 compute a SVM class probability for x \
   using K^test and the trained classifier;
end for
```

(b) Profile BPLA kernel

```
INPUT:
training data (set of RNA alignments)
test data (set of RNA alignments)

OUTPUT:
SVM class probability for each of test data


(1) Training

for each alignment X in training data
  P_x = COMPUTE_AVERAGED_BP_MATRIX(X);
  for each column i in X
    compute a base-pairing profile {P_x^L(i), P_x^R(i), P_x^U(i)};
  end for
end for

for each alignment X in training data
  for each alignment Y in training data
    compute a value of Profile BPLA kernel K^train(X,Y);
  end for
end for

Train a SVM classifier using K^train;


(2) Test

for each alignment X in test data
  P_x = COMPUTE_AVERAGED_BP_MATRIX(X);
  for each column i in X
    compute a base-pairing profile {P_x^L(i), P_x^R(i), P_x^U(i)};
  end for
end for

for each alignment X in test data
  for each alignment Y in training data
    compute a value of Profile BPLA kernel K^test(X,Y);
  end for
end for

for each alignment X in test data
 compute a SVM class probability for X \
   using K^test and the trained classifier;
end for

function COMPUTE_AVERAGED_BP_MATRIX(X)
  for each sequence X^k in X
    compute a base-pairing probability matrix P'_x^k;
  end for
  return the averaged matrix of P'_x^k;
end function
```
